# Stability metrics for optic radiation tractography: Towards damage prediction after resective surgery

**DOI:** 10.1016/j.jneumeth.2017.05.029

**Published:** 2017-08-15

**Authors:** Stephan Meesters, Pauly Ossenblok, Louis Wagner, Olaf Schijns, Paul Boon, Luc Florack, Anna Vilanova, Remco Duits

**Affiliations:** a Academic Center for Epileptology Kempenhaeghe & Maastricht University Medical Center, Netherlands; b Department of Mathematics & Computer Science, Eindhoven University of Technology, Netherlands; c Department of Biomedical Engineering, Eindhoven University of Technology, Netherlands; d Department of Neurosurgery, Maastricht University Medical Center, Netherlands; e Department of Mathematics and Computer Science, Delft University of Technology, Netherlands

**Keywords:** Optic radiation, Meyer's loop, Diffusion magnetic resonance imaging, Fiber tractography, Epilepsy, Neurosurgery

## Abstract

**Background:**

An accurate delineation of the optic radiation (OR) using diffusion MR tractography may reduce the risk of a visual field deficit after temporal lobe resection. However, tractography is prone to generate spurious streamlines, which deviate strongly from neighboring streamlines and hinder a reliable distance measurement between the temporal pole and the Meyer's loop (ML-TP distance).

**New method:**

Stability metrics are introduced for the automated removal of spurious streamlines near the Meyer's loop. Firstly, fiber-to-bundle coherence (FBC) measures can identify spurious streamlines by estimating their alignment with the surrounding streamline bundle. Secondly, robust threshold selection removes spurious streamlines while preventing an underestimation of the extent of the Meyer's loop. Standardized parameter selection is realized through test–retest evaluation of the variability in ML-TP distance.

**Results:**

The variability in ML-TP distance after parameter selection was below 2 mm for each of the healthy volunteers studied (*N* = 8). The importance of the stability metrics is illustrated for epilepsy surgery candidates (*N* = 3) for whom the damage to the Meyer's loop was evaluated by comparing the pre- and post-operative OR reconstruction. The difference between predicted and observed damage is in the order of a few millimeters, which is the error in measured ML-TP distance.

**Comparison with existing method(s):**

The stability metrics are a novel method for the robust estimate of the ML-TP distance.

**Conclusions:**

The stability metrics are a promising tool for clinical trial studies, in which the damage to the OR can be related to the visual field deficit that may occur after epilepsy surgery.

## Highlights

• The alignment of streamlines is quantified by fiber-to-bundle coherence measures.
• Reliable ML-TP distance measurement by removal of spurious (deviating) streamlines.
• Parameter estimation to remove spurious streamlines and to retain the Meyer's loop.
• The validity of ML-TP distance is estimated by pre and postoperative OR comparisons.
• The stability metrics are promising to relate OR damage to a visual field deficit.

## 1 Introduction

With diffusion tensor imaging (DTI) the morphology of brain tissue, and especially the white matter fiber bundles, can be investigated in vivo (Mori, 2007), offering new possibilities for the evaluation of brain disorders and preoperative counseling. The optic radiation (OR) is a collection of white matter fiber bundles which carries visual information from the thalamus to the visual cortex (Rubino et al., 2005). Numerous studies (Yogarajah et al., 2009, Taoka et al., 2005, Chen et al., 2009, Winston et al., 2012, Borius et al., 2014, James et al., 2015) have accomplished to reconstruct the OR with DTI, by tracking pathways between the lateral geniculate nucleus (LGN) and the primary visual cortex. In the curved region of the OR, configurations with multiple fiber orientations appear, such as crossings, because white matter tracts of the temporal stem intermingle with the fibers of the Meyer's loop (Kier et al., 2004). Therefore, it is especially challenging to reconstruct the Meyer's loop, which is the most vulnerable bundle of the OR in case of surgical treatment of epilepsy in which part of the temporal lobe is removed (James et al., 2015). However, a limitation of DTI is that it can extract only a single fiber direction from the diffusion MRI data.

**Fig. 1:**
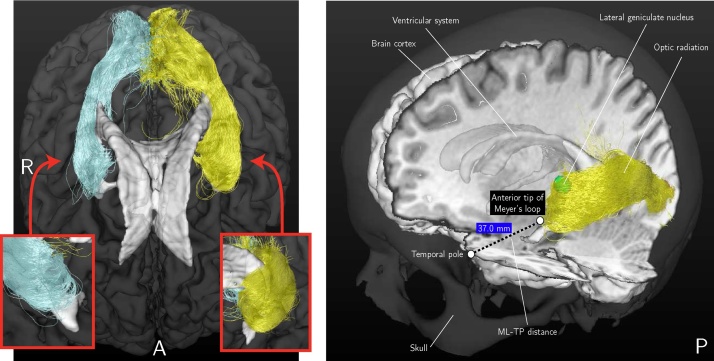
Left: An example of the reconstruction result of the OR using probabilistic tractography from an axial view. As inserts, close-ups are shown of the anterior tips of the reconstructions of the OR from a coronal view. Right: The tracking results are shown for the same volunteer in a composite image along with other brain structures such as the ventricular system. The ML-TP distance measurement is indicated.

With the advent of multi-fiber diffusion models it has become possible to describe regions of crossing fibers such as the highly curved Meyer's loop. Tractography based on constrained spherical deconvolution (CSD) (Tournier et al., 2007, Descoteaux et al., 2009) has been shown to have good fiber detection rates (Wilkins et al., 2015) and has been applied in several studies to reconstruct the OR (Lim et al., 2015, Martínez-Heras et al., 2015). Furthermore, probabilistic tractography is considered superior in comparison to deterministic tractography for resolving the problem of crossing fibers in the Meyer's loop (Lilja and Nilsson, 2015). The probabilistic tracking results between the LGN and the visual cortex for a healthy volunteer are illustrated in Fig. 1. The tracking results are shown in a composite image along with other brain structures such as the ventricular system.

However, a common occurrence in tractograms obtained from probabilistic tractography are spurious (deviating) streamlines. Spurious streamlines are by definition not well-aligned with neighboring streamlines and may hinder the measurement of the distance between the temporal pole to the tip of the Meyer's loop (ML-TP distance). An accurate measurement of the ML-TP distance is required for estimating the potential damage to the OR after temporal lobe resection (TLR). Methods have been proposed for the identification and removal of spurious streamlines, for example based on outlier detection (Yeatman et al., 2012, Martínez-Heras et al., 2015, Khatami et al., 2016), based on the prediction of diffusion measurements by whole-brain connectomics (Pestilli et al., 2014), or based on the uncertainty in the main eigenvector of the diffusion tensor (Parker et al., 2003). Most of these methods for reducing spurious streamlines are based on density estimation in ${\mathbb{R}}^{3}$. In contrast, in the current study fiber-to-bundle coherence (FBC) tractometry measures are employed that are based on density estimation in the space of positions and orientations ${\mathbb{R}}^{3}\times S^{2}$. The stability metrics introduced in this study are based on the FBC measures. These metrics provide a reliable OR reconstruction that is robust under stochastic realizations of probabilistic tractography. To achieve a reliable reconstruction of the full extent of the Meyer's loop, an appropriate selection of streamlines is required such that spurious streamlines are removed while preserving streamlines that are anatomically more likely to exist. For this purpose the FBC parameter *ϵ* is estimated based on the measured variability in ML-TP distance. Here we respect an a-priori constraint on the maximal ML-TP distance variability for a test–retest procedure on streamline tracking and determine the corresponding minimal threshold *ϵ*_selected_ on the FBC measures. This threshold removes a minimal amount of spurious streamlines while allowing for a stable estimation of the ML-TP distance.

In the current study the validity of the distance measurements is evaluated based on pre- and post-operative comparisons of the reconstructed OR of patients who underwent a TLR. It is investigated whether it is feasible to assess pre-operatively for each individual patient the potential damage to the OR as an adverse event of the planned TLR. The deviation between the prediction of the damage to the OR and the measured damage in a post-operative image is compared, giving an indication of the overall error in distance measurement.

The main contributions of this paper are:

• Quantification of spurious streamlines. We provide FBC measures that quantify how well-aligned a streamline is with respect to neighboring streamlines.
• Stability metrics for the standardized removal of spurious streamlines near the anterior tip of the Meyer's loop.
• Robust estimation of the variability in ML-TP distance by a test–retest evaluation.
• Demonstration of the importance of the FBC measures by retrospective prediction of the damage to the OR based on pre- and post-operative reconstructions of the OR of epilepsy surgery candidates.

## 2 Materials and methods

### 2.1 Subjects

Eight healthy volunteers without any history of neurological or psychiatric disorders were included in our study. All volunteers were male and in the age range of 21–25 years. Furthermore, three patients were included who were candidates for temporal lobe epilepsy surgery. For each patient a standard pre- and post-operative T1-weighted anatomical 3D-MRI was acquired. Patient 1 (46/F) was diagnosed with a right mesiotemporal sclerosis and had a right TLR, including an amygdalohippocampectomy. Patient 2 (23/F) was diagnosed with a left mesiotemporal sclerosis and had an extended resection of the left temporal pole. Lastly, Patient 3 (38/M) was diagnosed with a cavernoma located in the basal, anterior part of the left temporal lobe and had an extended lesionectomy. All patients had pre- and post-operative perimetry carried out by consultant ophthalmologists. The study was approved by the Medical Ethical Committee of Kempenhaeghe, and informed written consent was obtained from all subjects.

### 2.2 Data acquisition

Data was acquired on a 3.0 T magnetic resonance (MR) scanner, using an eight-element SENSE head coil (Achieva, Philips Health Care, Best, The Netherlands). A T1-weighted scan was obtained for anatomical reference using a Turbo Field Echo (TFE) sequence with timing parameters for echo time (TE = 3.7 ms) and repetition time (TR = 8.1 ms). A total of 160 slices were scanned with an acquisition matrix of 224 × 224 with isotropic voxels of 1 × 1 ×1 mm, leading to a field of view of 224 × 224 × 160 mm. Diffusion-weighted imaging (DWI) was performed using the Single-Shot Spin-Echo Echo-Planar Imaging (SE-EPI) sequence. Diffusion sensitizing gradients were applied, according to the DTI protocol, in 32 directions with a *b*-value of 1000 s/mm^2^ in addition to an image without diffusion weighting. A total of 60 slices were scanned with an acquisition matrix of 112 × 112 with isotropic voxels of 2 × 2 ×2 mm, leading to a field of view of 224 × 224 × 120 mm. A SENSE factor of 2 and a halfscan factor of 0.678 were used. Acquisition time was about 8 min for the DWI scan and 5 min for the T1-weighted scan. The maximal total study time including survey images was 20 min.

### 2.3 Data preprocessing

**Fig. 2:**
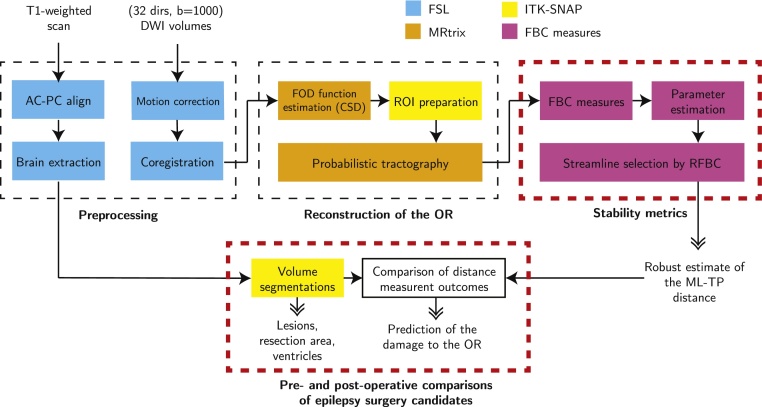
A schematic overview of the analysis procedures followed to reconstruct the optic radiation (OR) and to acquire a robust estimate of the Meyer's Loop to Temporal Pole (ML-TP) distance. The stages in which data are processed are indicated by the dashed boxes. The red dashed boxes indicate the new contributions of the study. The various software packages are color-coded. The inputs of the pipeline are a diffusion-weighted imaging (DWI) dataset and an anatomical T1-weighted MRI image. Outputs of the pipeline are shown with double-headed arrows. Abbreviations: FOD, fiber orientation density; CSD, constrained spherical deconvolution; ROI, region of interest; FBC, fiber to bundle coherence; RFBC, relative FBC. (For interpretation of the references to color in this figure legend, the reader is referred to the web version of this article.)

The preprocessing of the T1-weighted scan and DWI data is outlined in Fig. 2 (top-left box). All data preprocessing is performed using a pipeline created with NiPype (Gorgolewski et al., 2011), which allows for large-scale batch processing and provides interfaces to neuroimaging packages (FSL, MRtrix). The T1-weighted scan was first aligned to the AC-PC axis by affine coregistration (12 degrees-of-freedom) to the MNI152 template using the FMRIB Software Library v5.0 (FSL) (Jenkinson et al., 2012). Secondly, affine coregistration, considered suitable for within-subject image registration, was applied between the DWI volumes to correct for motion. Eddy current induced distortions were corrected within the Philips Achieva scanning software and did not require further post-processing. The DWI b=0 volume was subsequently affinely coregistered to the axis-aligned T1-weighted scan using normalized mutual information, and the resulting transformation was applied to the other DWI volumes. The DWI volumes were resampled using linear interpolation. After coregistration, the diffusion orientations were reoriented using the corresponding transformation matrices (Leemans and Jones, 2009).

### 2.4 Probabilistic tractography

Probabilistic tractography of the OR (outlined in Fig. 2, top-middle box) is based on the Fiber Orientation Density (FOD) function, first described by Descoteaux et al. (2009). With probabilistic tractography, streamlines are generated between two regions of interest (ROIs): the LGN, located in the thalamus, and the primary visual cortex (see Fig. 1). The LGN was defined manually on the axial T1-weighted image using anatomical references (lateral and caudal to the pulvinar of the thalamus) (Fujita et al., 2001) using a sphere of 4 mm radius, corresponding to a volume of 268 mm^3^. The ipsilateral primary visual cortex was manually delineated on the axial and coronal T1-weighted image. The primary visual cortex ROI's used in this study have an average volume of 1844 mm^3^.

The FOD function describes the probability of finding a fiber at a certain position and orientation (Tuch, 2004). In the current study the FOD function is estimated using CSD, which is implemented in the MRtrix software package (Tournier et al., 2012). During tracking, the local fiber orientation is estimated by random sampling of the FOD function. In the MRtrix software package, rejection sampling is used to sample the FOD function in a range of directions restricted by a curvature constraint imposed on the streamlines. Streamlines are iteratively grown until no FOD function peak can be identified with an amplitude of 10% of the maximum amplitude of the FOD function (Jeurissen et al., 2011, Tournier et al., 2012). In MRtrix tracking, 20,000 streamlines are generated, which provides a good balance between computation time and reconstruction ability. A step size of 0.2 mm and a radius of curvature of 1 mm were used. These settings are reasonable for our application of reconstructing the OR and are recommended by Tournier et al. (2012). The FOD function was fitted with six spherical harmonic coefficients, which is suitable for the DTI scanning protocol used in this study.

Anatomical constraints are applied when reconstructing the OR in order to prevent the need for manual pruning of streamlines and to reduce a subjective bias. Firstly, streamlines are restricted within the ipsilateral hemisphere. Secondly, fibers of the OR are expected to pass over the temporal horn of the ventricular system (Sincoff et al., 2004). The ventricular system is manually delineated using ITK-SNAP image segmentation software (Yushkevich et al., 2006). Streamlines that cross through the area superior-laterally to the temporal horn are retained. Thirdly, an exclusion ROI is created manually of the fornix to remove streamlines that cross this region, which is in close proximity to the LGN and Meyer's loop. Furthermore, in order to remove long anatomically implausible streamlines, the maximum length of the streamlines is set to 114 mm based on a fiber-dissection study of the OR by Peltier et al. (2006).

### 2.5 Quantification of spurious streamlines

The stability metrics to identify spurious streamlines are outlined in Fig. 2, top-right box. These metrics are used to provide a reconstruction of the OR that is robust against the presence of spurious streamlines, which occur especially near the anterior tip of the Meyer's loop as shown in Fig. 1 (left). The application of these metrics is important to obtain a stable measurement of the ML-TP distance as indicated in Fig. 1 (right).

**Fig. 3:**
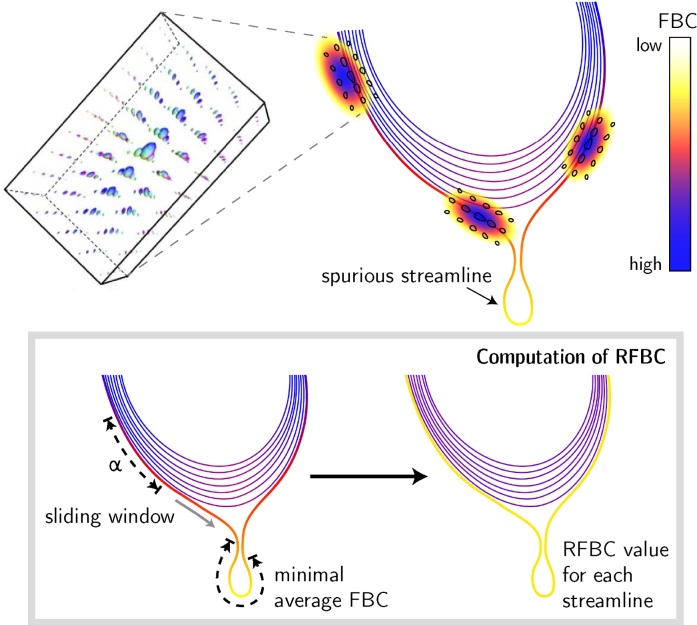
Top: The *fiber-to-bundle coherence* FBC measure is determined via kernel density estimation. A Brownian motion kernel is used (shown left), which is defined on the space of positions and orientations. The streamlines are color-coded according to their FBC measure, scaled from high (blue) to low (white). Bottom: The RFBC is computed using a sliding window of size *α* and produces a single value for each streamline. (For interpretation of the references to color in this figure legend, the reader is referred to the web version of this article.)

The *Fiber-to-Bundle Coherence FBC* measure, providing the basis of the stability metrics, is a quantitative measure of streamline alignment and is used for removing spurious streamlines. Spurious streamlines are (partially) poorly aligned with surrounding streamlines in the streamline bundle, which is illustrated schematically in Fig. 3 (top right). In order to compute the FBC, streamlines are lifted to 5D curves by including the local orientation of the tangent to the streamline. A lifted streamline *γ*_*i*_ can be written as

(1) $$\gamma_{i}=\left\{({\text{y}}_{i}^{k},{\text{n}}_{i}^{k})\in {\mathbb{R}}^{3}\times S^{2}\;|\;k=1,\ldots ,N_{i}\right\},$$

where **y** and **n** are the position and orientation of a streamline element, *N*_*i*_ is the number of points in the streamline and *i* denotes the index within the streamline bundle $\Gamma =\cup_{i=1}^{N}\{\gamma_{i}\}$. To include a notion of alignment between neighboring streamline tangents, we embed the lifted streamlines into the differentiable manifold of the rigid-body motion Lie group SE(3). Within this differential structure, a measure is defined that quantifies the alignment of any two lifted streamline points with respect to each other in the space of positions and orientations ${\mathbb{R}}^{3}\times S^{2}$ (Mumford, 1994, Citti and Sarti, 2006, Duits et al., 2007). In order to compute this measure, kernel density estimation is applied using a (hypo-elliptic) Brownian motion kernel (see Fig. 3, top left). The kernels used in the kernel density estimation have a probabilistic interpretation: they are the limiting distribution of random walkers in ${\mathbb{R}}^{3}\times S^{2}$ that randomly move forward or backward, randomly change their orientation, but cannot move sideways (Duits and Franken, 2011, Portegies et al., 2015). The FBC measure results from evaluating the kernel density estimator along each element of all lifted streamlines, shown in Fig. 3 (top right) where the FBC is color-coded for each streamline.

A spurious streamline can be identified by a low FBC that occurs anywhere along its path. For this purpose, a scalar measure for the entire streamline is introduced, called the relative FBC (RFBC), which computes the minimum average FBC in a sliding window along the streamline *γ*_*i*_ ∈ Γ relative to the bundle Γ. The RFBC for a streamline *γ*_*i*_ is calculated according to

(2) $${\mathrm{RFBC}}^{\alpha }(\gamma_{i},\Gamma )=\frac{{\mathrm{AFBC}}^{\alpha }(\gamma_{i},\Gamma )}{\mathrm{AFBC}(\Gamma )}.$$

The numerator AFBC^*α*^(*γ*_*i*_, Γ) gives the minimum average FBC of any segment of length *α* along the streamline *γ*_*i*_. The denominator *AFBC*(Γ) is used for normalization and is the average FBC of all the streamlines in the bundle, computed over the entire length of each streamline. The segment length *α* was determined empirically as 2 mm (corresponding to 10 streamline points when using a stepsize of 0.2 mm), which is considered small enough to characterize local deviations of the streamline but contains enough streamline points for stable quantification of local FBC. For a formal definition of the numerator and denominator in Eq. (2), see Eqs. (A.5) and (A.6) in Appendix A, respectively. Further details regarding the implementation of FBC measures, which includes several optimization steps such as pre-computed lookup tables for the Brownian motion kernels, are available in Appendix B.

### 2.6 Standardized parameter selection

To control the removal of spurious streamlines the threshold parameter *ϵ* is introduced, which is defined as the lower bound criterion on RFBC that retains a streamline. More precisely, every streamline *γ*_*i*_ that meets the condition RFBC^*α*^(*γ*_*i*_, Γ) ≥ *ϵ* is retained. However, a careful selection of this threshold is required in order to prevent an underestimation of the full extent of the Meyer's loop. A method is introduced for the standardized selection of the minimal threshold *ϵ*_selected_ through test–retest evaluation of the variability in ML-TP distance. To this end, probabilistic tractography of the OR is performed multiple times, followed by the computation of the RFBC measure in each repetition. Subsequently, a parameter sweep is performed in which *ϵ* is varied between 0 ≤ *ϵ* ≤ *ϵ*_max_ where *ϵ*_max_ corresponds to the state where all streamlines are removed from Γ. During every step of the parameter sweep, the ML-TP distance is calculated for all test–retest repetitions by computing the Hausdorff distance (Rockafellar and Wets, 2005) between the temporal pole and the OR. Using these distance measurements, the mean and the standard deviation (variability) of the ML-TP distance are determined for each value of *ϵ*.

**Fig. 4:**
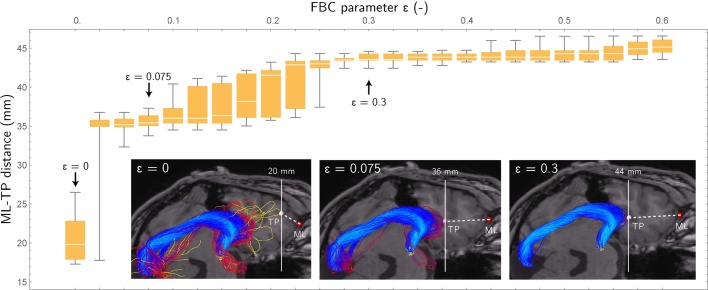
Boxplot showing the mean and standard deviation of the estimated ML-TP distances for test–retest evaluation of the reconstruction of the OR for an example healthy volunteer. A sweep from low to high *ϵ* is performed to evaluate the effect of removing streamlines on the stability of the estimated ML-TP distance.

The procedure is illustrated for a healthy subject in Fig. 4, showing the mean and standard deviation of the ML-TP distance for increasing values of *ϵ*. Initially, a high variability is seen at *ϵ* = 0, indicating the presence of spurious streamlines near the anterior tip of the Meyer's loop. At *ϵ* = 0.075 most spurious streamlines are removed and a variability in the order of several millimeters is seen. The variability rises and falls during 0.1 ≤ *ϵ* ≤ 0.3. A stable region is obtained at *ϵ* ≈ 0.3, however at this point too many streamlines have been discarded according to the condition RFBC^*α*^(*γ*_*i*_, Γ) ≥ *ϵ* and thereby the ML-TP distance will be overestimated. In order to estimate the minimal threshold *ϵ*_selected_, in which the ML-TP distance is neither under- nor overestimated, a maximum is set for the variability of 2 mm. This maximum is based on the maximal accuracy of 2–5 mm that may be achieved during resective surgery. In the selection procedure, *ϵ* is set at the first occurrence of low variability, i.e.

(3) $$\epsilon_{\mathrm{selected}}=\min\{\epsilon >0\;|\;\sigma (\epsilon )\leq 2\mathrm{mm},\sigma^{\prime }(\epsilon )=0,\sigma^{\prime \prime }(\epsilon )>0\}$$

where *σ*(*ϵ*) denotes the standard deviation in ML-TP for the chosen *ϵ*. After crossing the 2 mm threshold on variability, *ϵ*_selected_ is placed on the local minimum of *σ*(*ϵ*). Using this procedure, in the example shown in Fig. 4 the ML-TP is estimated for *ϵ* = 0.075 at 36 mm. This ML-TP distance is within the range of 22–37 mm as reported by Ebeling et al. (1988), who performed a dissection study on 25 human cadavers.

For the patients studied, the distance measurement outcomes are compared to the predicted damage of the OR after surgery, as outlined in Fig. 2 (bottom row, red dashed box). The resection area is manually delineated in the post-operative T1-weighted image using ITK-SNAP (Yushkevich et al., 2006). The resection length is measured from the temporal pole, at the anterior tip of the middle sphenoid fossa, up to the posterior margin of the resection. The predicted damage is determined by the distance between the pre-operative ML-TP distance and the resection length. The difference between the predicted damage and the observed damage, given by the distance between pre- and post-operative ML-TP distances, is named the margin of error. The margin of error indicates the maximal error in distance measurements, which includes both the variability in probabilistic tractography and unaccounted sources of error such as brain shift or distortions.

### 2.7 Open source software

The methodology for the robust reconstruction of the OR (outlined in Fig. 2) is available as an open source software package. The NiPype based pipeline for the basic processing of DW-MRI data, tractography, and FBC measures is available at https://github.com/stephanmeesters/DWI-preprocessing. An open source implementation of the FBC measures for the reduction of spurious streamlines described in Appendix A, Appendix B is available in the DIPY (Diffusion Imaging in Python) framework (Garyfallidis et al., 2014) or as a C++ stand-alone application at https://github.com/stephanmeesters/spuriousfibers. Visualization was performed in the open source vIST/e tool (Eindhoven University of Technology, Imaging Science & Technology Group, http://sourceforge.net/projects/viste/).

## 3 Results

### 3.1 Robust estimation of ML-TP distance

The effect of the removal of spurious streamlines on the ML-TP distance measurement using the FBC measures is demonstrated for eight healthy volunteers. For each volunteer the mean ML-TP distance and its standard deviation are listed in Table 1 for the left and right hemisphere, together with its corresponding test–retest variability. The additional value of the FBC measures for a robust ML-TP distance measurement is further evaluated for three patients who underwent a TLR.

**Table 1:** Listed are the ML-TP distances estimated for the left and right hemispheres of the healthy volunteers studied (*N* = 8) and the corresponding selected values for the FBC thresholding parameter *ϵ*.

Volunteer	ML-TP distance		*ϵ*	
	Left (mm)	Right (mm)	Left (–)	Right (–)
1	36.4 ± 1.5	32.1 ± 1.3	0.075	0.15
2	30.0 ± 0.6	27.8 ± 1.0	0.13	0.14
3	33.4 ± 1.5	23.5 ± 0.9	0.2	0.35
4	34.9 ± 1.7	31.4 ± 0.2	0.45	0.1
5	36.8 ± 1.4	32.2 ± 1.0	0.075	0.33
6	28.3 ± 0.3	25.8 ± 0.6	0.025	0.28
7	32.3 ± 0.4	23.4 ± 1.1	0.15	0.05
8	22.5 ± 0.5	30.7 ± 1.0	0.125	0.18

**Fig. 5:**
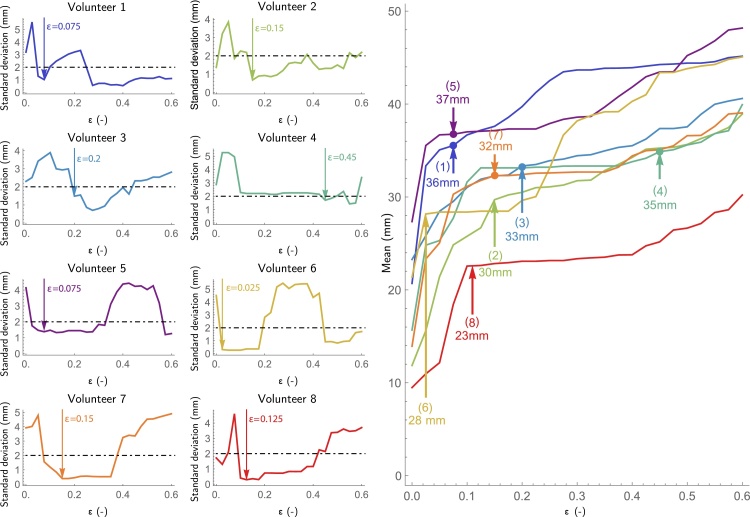
Shown is the parameter estimation for the reconstructed left OR of the eight healthy volunteers studied. Left: The standard deviation of the ML-TP distance is shown as a function of *ϵ*. For each healthy volunteer a suitable choice of *ϵ* is made at the point where the standard deviation first drops below the threshold of 2 mm and reaches a local minimum, shown by the black dotted line. Right: The estimated mean ML-TP distance is shown as a function of *ϵ*. The *ϵ*_selected_ for each volunteer is indicated by an upwards pointed arrow, indicated along with the values of the associated estimate of the ML-TP distance.

The parameter estimation based on test–retest evaluation is illustrated in Fig. 5 for the reconstructed OR of the left hemisphere for the eight healthy volunteers studied, showing for a range of parameter *ϵ* (0–0.6) the standard deviation (left) and the mean (right) of the estimated ML-TP distance. The test–retest evaluation was performed with 10 repeated tractograms of the OR, which was empirically determined to be a good balance between group size and computation time. For all volunteers evaluated, a high standard deviation of the ML-TP distance (over 2 mm) was observed at low values of *ϵ* (0.0–0.05), which indicates the presence of spurious streamlines with a very low RFBC. The corresponding mean ML-TP distance reflects large jumps for an increase of the value of *ϵ* from 0 to 0.05, showing an average increase for the eight healthy volunteers of 8 mm. For each healthy volunteer the *ϵ*_selected_ is selected according to Eq. (3). The *ϵ*_selected_ corresponds to a mean ML-TP distance that is depicted by the arrows in Fig. 5 (right) for the eight healthy volunteers studied. After the initial high variability of the ML-TP distance, a stable region occurred for all healthy volunteers in which the standard deviation was below 2 mm. The healthy volunteers 1, 5 and 4 indicated regions of instability for relatively high values of *ϵ*. This can be attributed to gaps within the reconstructed OR with a lower number of streamlines compared to the main streamline bundle. Lastly, it can be observed that for volunteer 4 the selected *ϵ* is large compared to the other healthy volunteers. However, for this volunteer the mean ML-TP distance is stable from *ϵ* = 0.15 onward and therefore does not reflect an overestimation of the ML-TP distance.

On the group level the ML-TP distances listed in Table 1 are on average 31.7 ± 4.7 mm for the left hemisphere and 28.4 ± 3.8 mm for the right hemisphere. The mean variability in probabilistic tractography on the individual level for the group of healthy volunteers is 1.0 mm and 0.9 mm for the left and right hemispheres, respectively. Large deviations in ML-TP distance were observed between the left and right hemispheres, especially, for volunteers 3, 7 and 8.

### 3.2 Pre- and post-operative comparisons

**Fig. 6:**
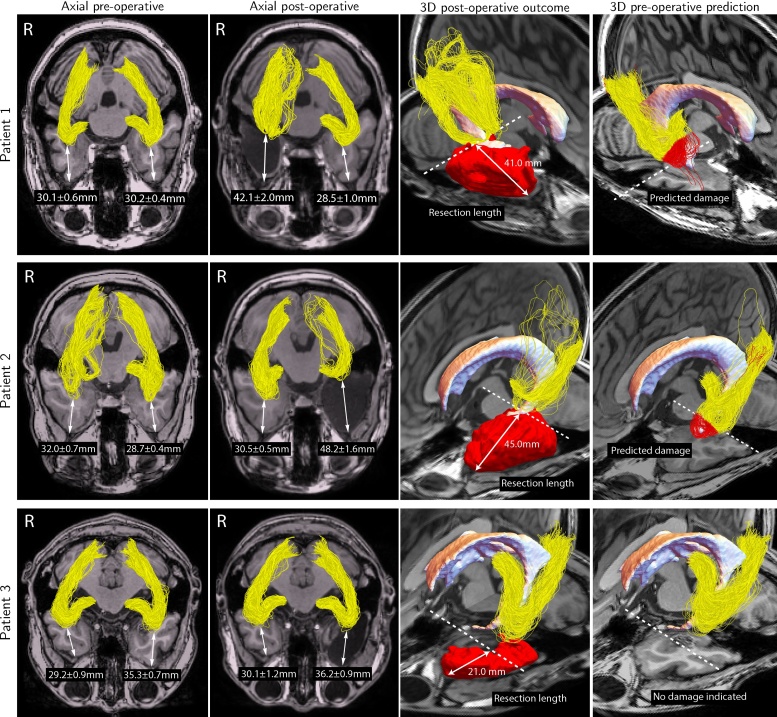
Tractography and distance measurement results for the three patients included in the study. The first and second columns show the reconstructions of the OR before and after surgery, respectively. For each reconstruction the ML-TP distance and associated variability are displayed. The third and fourth columns show a 3D view of the reconstruction of the OR in the affected hemisphere after and before surgery, respectively. The resection area is displayed in red and the predicted damage is indicated by color-coded red streamlines. (For interpretation of the references to color in this figure legend, the reader is referred to the web version of this article.)

The importance of the robust ML-TP distance measurement is illustrated for three patients who underwent resective epilepsy surgery. Fig. 6 displays the pre-operative (first and last columns) and post-operative reconstructions (second and third columns) of the OR and indicates for both hemispheres the estimated ML-TP distances (first and second column). Given is also the resection length (third column) and the pre-operative reconstruction of the OR along with the predicted damage, indicated by the red colored streamlines (fourth column). The pre- and post-operative distance measurements and the corresponding values of *ϵ* are listed for both the left and right hemisphere in Table 2. Furthermore, the predicted damage is listed in Table 2 and reflects the distance between the pre-operative ML-TP distance and the resection length. Finally, the margin of error is indicated, defined as the difference between the predicted damage and the observed damage.

**Table 2:** The results listed for the pre- and post-operative comparison of the reconstruction of the OR for both hemispheres of the three epilepsy surgery candidates included in our study. Distance measurements of the anterior extent of the OR to the temporal pole (ML-TP) are displayed along with the variability in probabilistic tractography for the corresponding *ϵ*_selected_. Furthermore, the resection lengths, predicted and observed damages, and the measured margins of error are listed for the affected hemispheres.

Patient/hemisphere	ML-TP distance		*ϵ*	Resection length (mm)	Predicted damage (mm)	Observed damage (mm)	Margin of error (mm)
	Pre-op (mm)	Post-op (mm)	Pre-op (–)/Post-op (–)				
*Patient 1*							
Left	30.2 ± 0.4	28.5 ± 1.0	0.10/0.20	–	–	–	–
Right	30.1 ± 0.6	42.1 ± 2.0	0.48/0.18	41.0	10.9 ± 0.6	12.0 ± 2.6	4.3

*Patient 2*							
Left	28.7 ± 0.4	48.2 ± 1.6	0.13/0.4	45.0	16.3 ± 0.4	19.5 ± 2.0	5.6
Right	32.0 ± 0.7	30.5 ± 0.5	0.13/0.2	–	–	–	–

*Patient 3*							
Left	35.3 ± 0.7	36.2 ± 0.9	0.10/0.18	21.0	0.0	0.0	1.6
Right	29.2 ± 0.9	30.1 ± 1.2	0.22/0.18	–	–	–	–

The tractography results indicate that for patients 1 and 2 the OR is damaged, likely resulting in a disrupted Meyer's loop for both patients. The perimetry results of these patients indicated a visual field deficit (VFD) of 60 degrees for patient 2, which was smaller than the VFD measured for patient 1 at 90 degrees despite the larger resection of patient 2 (see Table 2). Note, that for patient 3, for whom there was no damage to the OR, the reconstruction of the OR is well reproducible for both hemispheres, with a difference of maximally 3.0 mm including the variability in ML-TP distance. The difference between the predicted damage and the observed damage was small for these patients, indicating an maximum error of the predicted damage of the OR of 5.6 mm or less. The reproducibility of the reconstruction results obtained following the procedures as here described is further confirmed by the unaffected hemispheres of each individual patient, which show a similar anterior extent for both pre- and post-operative reconstructions of the OR. The ML-TP distance of the OR reconstructed for the OR of the non-pathologic hemisphere showed deviations for the two different scans of maximally 3.1 mm, 2.7 mm and 3.0 mm for Patient 1, Patient 2 and Patient 3, respectively, including the variability measure. The overall mean ML-TP distance pre-operatively is 31.4 ± 3.5 mm for the left hemisphere and 30.4 ± 1.4 mm for the right hemisphere. The mean variability in probabilistic tractography is 0.5 mm and 0.7 mm for the left and right hemispheres, respectively.

## 4 Discussion

Stability metrics were introduced for a robust estimation of the distance between the tip of the Meyer's loop and temporal pole. Standardized removal of spurious fibers was achieved, firstly by quantification of spurious streamlines using the FBC measures, and secondly by a procedure for the automatic selection of the minimal threshold *ϵ*_selected_ on the FBC measures. The results presented indicate that a reliable localization of the tip of the Meyer's loop is possible and that it is feasible to predict the damage to the OR as result of a TLR performed to render patients seizure free.

### 4.1 Procedures for the reconstruction of the OR

For the estimation of the FOD function, CSD was applied on diffusion data obtained with the prevalent DTI acquisition scheme, thus allowing for a broad clinical applicability. In the current study, the DTI acquisition scheme (*b* = 1000, 32 directions) has a relatively low number of directions of diffusion. Since the tip of the Meyer's loop has a high curvature, its reconstruction could especially benefit from the HARDI acquisition scheme (Tuch et al., 2002), which measures a larger number of directions of diffusion such as 64 or 128 directions. However, unlike DTI, HARDI is not commonly applied within a medical MRI diagnosis. Instead, the DTI data may be improved by applying contextual enhancement (Tax et al., 2014, Portegies et al., 2015), such as the one available in the DIPY framework (http://dipy.org). Additionally, in order to improve the image quality of the diffusion measurements it may be beneficial to apply denoising. This may, for example, be achieved by a recently proposed denoising approach based on non-local principal component analysis (PCA) (Manjon et al., 2015).

The MRtrix software package was employed for the estimation of the FOD function and for performing probabilistic tractography. As an alternative to the rejection sampling method that is implemented in MRtrix for sampling the FOD during tracking, the importance sampling method as introduced in Friman et al. (2006) could be used. In contrast to the hard constraints used in rejection sampling, the importance sampling method provides a soft constraint on the space of positions and orientations, which is in line with the mathematical framework introduced in this paper (see Appendix A).

The seed regions of the LGN and visual cortex are highly influential for the tractography results (Lilja et al., 2016). It may be possible to improve the fiber orientation estimation at the white matter to gray matter interface, such as near the LGN and visual cortex ROIs, by applying the recently introduced informed constrained spherical deconvolution (iCSD) (Roine et al., 2015). iCSD improves the FOD by modifying the response function to account for non-white matter partial volume effects, which may improve the reconstruction of the OR. In the current study, the LGN was identified manually and could possibly be improved by using a semi-automatic method such as presented by Winston et al. (2011). Another approach proposed by Benjamin et al. (2014) is to place different ROIs around the LGN and within the sagittal stratum, or by seeding from the optic chiasm (Kammen et al., 2016). A recent study suggested using seeding around the Meyer's loop with an a-priori fiber orientation (Chamberland et al., 2017).

### 4.2 Application of the stability metrics

The FBC measures are used for the quantification of spurious streamlines. These FBC measures are based on the estimation of streamline density in the space of positions and orientations ${\mathbb{R}}^{3}\times S^{2}$. An advantage of the FBC method is that it is generally applicable, regardless of the type of diffusion model and the tracking algorithm being used, since it depends only on the outcome of tractography. A possible limitation of the FBC measures are the number of streamlines that can be processed, since for densely populated regions of streamlines the method is computationally expensive. However, through the use of several optimization steps such as pre-computed lookup tables for the Brownian motion kernel, multi-threaded processing, subsampling of streamlines, and the exclusion of far-away streamline points, the computation times maintain manageable. Details are available in Appendix B.

In order to remove spurious fibers while preventing an underestimation of the full extent of the Meyer's loop, a procedure for estimating *ϵ*_selected_ was introduced based on the test–retest evaluation of the variability in ML-TP distance. Using this methodology, a robust measurement of the ML-TP distance was achieved in the left and right hemispheres of eight healthy volunteers. The variability in the reconstruction results of the OR stems mostly from data acquisition (e.g. SNR, partial volume effects, and patient motion) (Wakana et al., 2007). Therefore, *ϵ*_selected_ may vary between pre- and post-operative scans in the non-affected hemisphere (see Table 2). The mean ML-TP distances for both brain hemispheres, measured to be 30.0 ± 4.5 mm for the healthy volunteer group and 30.9 ± 2.4 mm for the patient group (pre-operatively), are within the range of the ML-TP distance reported on by Ebeling et al. (1988) and outcomes from other OR reconstruction methodologies. For example, ConTrack (Sherbondy et al., 2008) showing 28 ± 3.0 mm, Streamlines Tracer technique (STT) showing 37 ± 2.5 mm (Yamamoto et al., 2005) and 44 ± 4.9 mm (Nilsson et al., 2007), Probability Index of Connectivity (PICo) showing 36.2 ± 0.7 mm (Dayan et al., 2015), tractography on Human Connective Project (HCP) multi-shell data showing 30.7 ± 4.0 mm (Kammen et al., 2016), and MAGNET showing 36.0 ± 3.8 mm (Chamberland et al., 2017). It appeared, furthermore, that the mean ML-TP for both the healthy volunteers and the patients was larger in the left hemisphere compared to the right hemisphere, which is not consistent with a recent study by James et al. (2015) that indicated a significantly higher ML-TP in the right hemisphere.

A possible limitation of the parameter estimation procedure is that its application is tailored towards OR tractography. Unlike the FBC measures, which can be used for any tractogram, the parameter estimation procedure may not be generally applicable for other fiber bundles since a distance measurement between well-defined landmarks is required. However, a possible approach for generalized parameter selection is to fit the streamline bundle on a manifold such as used by BundleMAP (Khatami et al., 2016) and optimize *ϵ*_selected_ by minimizing the spread on the manifold.

### 4.3 Towards damage prediction for epilepsy surgery

The methodology for the estimation of the ML-TP distance is applied for the surgical candidates, firstly to assess the validity of the distance measurements, and secondly to indicate its additional value for resective epilepsy surgery. An indication of the validity of distance measurements was given by the margin of error, which was the largest for patient 2 amounting to 5.6 mm. The margin of error observed for the three patients can be lowered, e.g. by correcting for brain shifts that occur due to resection and CSF loss (Warfield et al., 2005) and by correcting for distortions present in MR echo-planar imaging (Jezzard and Balaban, 1995, Holland et al., 2010). The measurement of the ML-TP distance may be further complicated due to a shifted location of the temporal pole, or even its complete absence. However, the reproducibility of the pre- and post-operative reconstructions of the OR in the non-pathological hemisphere indicates that the effects of brain shift and imaging distortions may be limited. Small deviations in the ML-TP distance were seen (see Table 2), which suggests a good reproducibility, albeit for a limited number of patients.

In the standardized estimation procedure of *ϵ*_selected_ the maximal variability was set at 2 mm, both for the OR reconstructions of the healthy volunteers and the patients, which is based on the maximal surgical accuracy that can be achieved during standard or tailored anterior temporal lobectomy before the leakage of cerebrospinal fluid (CSF). A surgical accuracy below 2 mm has been reported (Tibbals, 2010) if a stereotactic frame is used or robotic assistance is involved. After the leakage of CSF however, cortical displacement up to 24 mm may be seen (Hastreiter et al., 2004), while other sources of inaccuracy are likely present such as echo-planar imaging distortion, partial volume effects, and image noise. However, despite these inaccuracies the pre- and post-operative comparison of the OR reconstructions indicates that the procedures developed in this study are a valid tool to assess the robustness of the distance measurements.

It appeared that the robust estimation of the ML-TP distance enabled to predict the damage of the OR after surgery, which was concordant with the actual damage for the three patients studied. Based on the damage prediction the margin of error was estimated, giving an indication of the overall error in distance measurement. The perimetry results of two of the patients studied indicated damage of either the left or right visual field, corresponding to a disruption of the Meyer's loop. A relatively small VFD was indicated for patient 2 despite the large temporal lobe resection. This result may be indicative of the large inter-patient variability in OR anatomy and function, but may also be the result of the non-standardized procedures for visual field testing in-between hospitals. It is recommended to evaluate the developed methodology further in a clinical trial including a sizable group of patients who are candidate for a TLR in order to be able to assess what the relation is between a VFD and the damage to the OR after a TLR.

## 5 Conclusion

It was shown for a group of healthy volunteers included in this study that standardized removal of spurious streamlines provides a reliable estimation of the distance from the tip of the Meyer's loop to the temporal pole that is stable under the stochastic realizations of probabilistic tractography. Pre- and post-operative comparisons of the reconstructed OR indicated, furthermore, (1) the validity of a robust ML-TP distance measurement to predict the damage to the OR as result of resective surgery, and (2) the high reproducibility of the reconstructions of the non-pathological hemisphere. In conclusion, the developed methodology based on diffusion-weighted MRI tractography is a step towards applying optic radiation tractography for pre-operative planning of resective surgery and for providing insight in the possible adverse events related to this type of surgery.

## Conflicts of interest

The authors declare that the research was conducted in absence of any commercial or financial relationships that could be construed as a possible conflict of interest.

## Appendix A Mathematical background

The fiber-to-bundle coherence (FBC) measures are based on kernel density estimation in the non-flat 5D position-orientation domain. First of all, each equidistantly sampled streamline $\gamma_{i}=\left\{\gamma_{i}^{k}\;|\;k=1,\ldots ,N_{i}\right\}$ with $\gamma_{i}^{k}=({\text{y}}_{i}^{k},{\text{n}}_{i}^{k})\in {\mathbb{R}}^{3}\times S^{2}$ are represented by delta distributions $\delta_{\left({\text{y}}_{i}^{j},{\text{n}}_{i}^{j}\right)}$. Here, *N*_*i*_ denotes the number of streamline points of streamline *γ*_*i*_ and ${\text{y}}_{i}^{k}$ and ${\text{n}}_{i}^{k}$ denote the position and tangent orientation of the streamline point $\gamma_{i}^{k}$, respectively. The full lifted output of the tractography is given by

(A.1) $$F_{\Gamma }(\text{y},\text{n})=\frac{1}{N_{\mathrm{tot}}}\sum_{\sigma =1}^{2}\sum_{i=1}^{N_{\mathrm{tot}}}\sum_{j=1}^{N_{i}}\delta_{\left({\text{y}}_{i}^{j},{\left(-1\right)}^{\sigma }{\text{n}}_{i}^{j}\right)}(\text{y},\text{n}),$$

where $\Gamma =\cup_{i=1}^{N_{\mathrm{tot}}}\{\gamma_{i}\}$ denotes the streamline bundle and *N*_tot_ indicates the number of streamlines in the bundle Γ. The summation over *σ* is used to include antipodal symmetry (where we identify ${\text{n}}_{i}^{j}∼-{\text{n}}_{i}^{j}$) of each tangent orientation ${\text{n}}_{i}^{j}$.

The kernel density estimator is defined by Fokker-Planck diffusion equations, which describe Brownian motion on ${\mathbb{R}}^{3}⋊S^{2}$ (Øksendal, 2003, Duits and Franken, 2011, Duits et al., 2013). The following evolution process is used where *F* = *F*_Γ_ serves as the initial condition,

(A.2) $$\left\{\begin{array}{c} \partial_{t}W_{F}(\text{y},\text{n},t)=(D_{\mathrm{spat}}{\left(\text{n}\cdot \nabla_{\text{y}}\right)}^{2}+D_{\mathrm{ang}}\Delta_{S^{2}})W_{F}(\text{y},\text{n},t),\\ W_{F}(\text{y},\text{n},0)=F(\text{y},\text{n}). \end{array}\right)$$

Here, *t* ≥ 0 is the evolution time, *D*_spat_ > 0 is the coefficient for spatial smoothing strictly in the direction of **n**. *D*_ang_ is the coefficient for angular smoothing ($\Delta_{S^{2}}$ is the Laplace-Beltrami operator on the sphere *S*^2^). In this evolution process, *W*_*F*_(**y**, **n**, *t*) represents the transition density of a moving particle with position **y** and orientation **n** at the time *t* ≥ 0, given that it started with initial distribution *F*(**y**, **n**) at *t* = 0. Then, the Local FBC (LFBC) is the result of evaluating the Brownian motion kernel *p*_*t*_ (see Fig. 3, left) along each element of the lifted streamline

(A.3) $$\begin{array}{c} \mathrm{LFBC}(\text{y},\text{n},\Gamma )=(p_{t}{*}_{{\mathbb{R}}^{3}\times S^{2}}F)(\cdot )=\int_{{\mathbb{R}}^{3}}\int_{S^{2}}p_{t}(R_{{\text{n}}^{\prime }}^{T}(\text{y}-{\text{y}}^{\prime }),R_{{\text{n}}^{\prime }}^{T}\text{n})F({\text{y}}^{\prime },{\text{n}}^{\prime })d\sigma ({\text{n}}^{\prime })d{\text{y}}^{\prime }. \end{array}$$

Here, *p*_*t*_(**y**, **n**) denotes the Green's function of the evolution process in Eq. (A.2), which equals the probability density of finding a random oriented particle at position **y**, with orientation **n**, at time *t* ∈ *R*^+^ given that it started at position **0** and orientation **e_z_** ∈ *S*^2^ at time 0. Likewise, $p_{t}(R_{{\text{n}}^{\prime }}^{T}(\text{y}-{\text{y}}^{\prime }),R_{{\text{n}}^{\prime }}^{T}\text{n})$ is the probability density of finding a random oriented particle at position **y** and orientation **n** given that it started at position **y**′ and orientation **n**′ at *t* = 0. Here, *dσ* is the usual measure on the sphere *S*^2^. As a result, by superposition in (A.3), LFBC(**y**, **n**, Γ) denotes the probability density of finding a random oriented particle at **y** and pointing at orientation **n** at time *t* > 0 given that it started at some point of the bundle Γ at *t* = 0. For exact formulas for the kernel *p*_*t*_(**y**, **n**), and the Gaussian approximations that we used for our computations, see Portegies et al. (2015).

A whole streamline measure, the relative FBC (RFBC), is calculated by the minimum of the moving average LFBC along the streamline *γ*_*i*_

(A.4) $${\mathrm{RFBC}}^{\alpha }(\gamma_{i},\Gamma )=\frac{{\mathrm{AFBC}}^{\alpha }(\gamma_{i},\Gamma )}{\mathrm{AFBC}(\Gamma )},$$

where AFBC^*α*^(*γ*_*i*_, Γ) indicates the minimal average LFBC over a small segment of the streamline with length *α*, given by

(A.5) $${\mathrm{AFBC}}^{\alpha }(\gamma_{i},\Gamma )=\min_{a\in [0,l_{i}-a]}\frac{1}{\alpha }\int_{a}^{a+\alpha }\mathrm{LFBC}(\gamma_{i}(s),\Gamma )\mathrm{ds},$$

and AFBC(Γ) is the average FBC of the entire streamline bundle Γ

(A.6) $$\mathrm{AFBC}(\Gamma )=\frac{1}{N}\sum_{i=1}^{N}\mathrm{FBC}(\gamma_{i},\Gamma ),$$

where

(A.7) $$\mathrm{FBC}(\gamma_{i},\Gamma )=\frac{1}{l_{i}}\int_{0}^{l_{i}}\mathrm{LFBC}(\gamma_{i}(s),\Gamma )\mathrm{ds}.$$

Here, *l*_*i*_ is the total length of the spatially projected curve **x**_*i*_(·) of fiber *γ*_*i*_ = (**x**_*i*_, **n**_*i*_). Further details of the computation of FBC can be found in Portegies et al. (2015).

## Appendix B Computational optimization

The FBC measures are implemented inside DIPY (Garyfallidis et al., 2014) using the high-speed Cython (C++ in Python) language. The kernel density estimation is executed with multithreading via the OpenMP library, which especially for cluster computing provides a significant speedup. To further accelerate the kernel density estimation, lookup-tables are computed containing rotated versions of the kernel *p*_*t*_ rotated over a discrete set of orientations (Rodrigues et al., 2010). The rotated versions are equally distributed over a sphere to ensure rotationally invariant processing. To be able to use the lookup table during kernel density estimation, each (continuous) streamline tangent orientation is matched with the closest (discrete) orientation on the sphere. For efficient implementation of orientation matching, a KD-tree is used, which is a multi-dimensional (*K* = 3) binary space partitioning, to minimize the number of angular distance computations.
